# Exploring the Relationship Between Energy Poverty and Health: A Pilot Study in Valencia

**DOI:** 10.3390/healthcare13243238

**Published:** 2025-12-10

**Authors:** Belén Costa-Ruiz, Maite Ferrando-García, Elena Rocher, Pilar Jordà

**Affiliations:** 1 Kveloce (Senior Europa SL), Plaza de la Reina, 19, 46033 Valencia, Spain; mferrando@kveloce.com; 2 Valencia Innovation Capital, Calle Juan Verdeguer, 16, 46024 Valencia, Spain; 3 Fundación Valencia Clima i Energía, Calle Juan Verdeguer, 16, 46024 Valencia, Spain; pilar.jorda@climaienergia.com

**Keywords:** energy poverty, health outcomes, mental and physical health, thermal comfort, vulnerable populations, social determinants of health, Mediterranean contexts, public health

## Abstract

**Background**: Energy poverty has emerged as a major societal challenge in Europe. **Objectives**: This study provides evidence on how different dimensions of energy poverty affect specific health outcomes, informing both theoretical understanding and intervention development to address this critical public health issue. **Methods**: We conducted a cross-sectional analysis using baseline data from the Valencia pilot of the WELLBASED project, examining associations between energy poverty dimensions and health outcomes among 322 vulnerable participants in Valencia, Spain (69.6% women, mean age 48.8 years). Data were collected through validated instruments, including EQ-5D-5L, DASS-21, and SF-12, alongside standardised energy poverty indicators developed by the Energy Poverty Advisory Hub (EPAH). **Results**: Energy poverty prevalence was notably high, with 69.9% of participants unable to maintain adequate warmth during winter and 72.4% experiencing cooling difficulties during summer. Statistical analyses revealed significant associations between energy poverty indicators and health outcomes. For example, mental health impacts were particularly pronounced, with thermal inadequacy associated with depression, anxiety, and stress (effect sizes eta2 = 0.042–0.126). Physical health showed condition-specific patterns: respiratory conditions linked to heating inadequacy, cardio-vascular conditions to cooling inadequacy, and musculoskeletal conditions to utility bill arrears. Participants with arrears on energy bills reported significantly higher chronic disease burden compared to those without arrears (3.08 vs. 2.40, *p* = 0.010). **Conclusions**: These findings suggest that addressing energy poverty is essential for health equity strategies. Urban contexts with Mediterranean climate patterns present unique challenges, re-quiring year-round interventions that address both winter heating and summer cooling, moving beyond the traditional cold-weather focus.

## 1. Introduction

Energy poverty has emerged as a major societal challenge in Europe, affecting millions of people and substantially compromising their health and quality of life [1]. The European Union defines energy poverty as “any situation in which a household cannot access essential energy services that provide basic levels and decent standards of living and health, including adequate heating, hot water, cooling, lighting, and energy to power appliances, in the relevant national context, existing social policy and other relevant policies, caused by a combination of factors, including but not limited to non-affordability, insufficient disposable income, high energy expenditure and poor energy efficiency of homes” [2]. This definition places health and well-being at the centre of the conceptualisation of energy poverty, recognising that inadequate access to energy services extends beyond economic hardship to become a fundamental determinant of health outcomes.

This relationship between energy poverty and health operates through multiple interconnected pathways, creating a complex system of physical, mental, and social health impacts. Research has demonstrated that inadequate indoor temperatures—whether excessive cold or heat—have deep implications across different health outcomes. Liddell and Morris (2010) conducted a comprehensive review establishing that cold housing increases the risk of cardiovascular disease, respiratory illness, and excess winter mortality, particularly among vulnerable populations [3]. The Marmot Review team (2011) identified that the health effects occur through different mechanisms; for example, cold stress increases blood pressure and exacerbates chronic diseases such as arthritis, asthma, and chronic obstructive pulmonary disease [4].

Furthermore, the mental health consequences of energy poverty are increasingly recognised as significant and widespread. Thomson et al. (2017) found significant associations between cold housing and increased risk of anxiety and depression across 32 European countries [5]. The psychological burden manifests through multiple mechanisms that interact and compound one another. Liddell and Guiney (2015) developed frameworks for understanding how living in cold and damp homes impacts mental well-being, identifying pathways including the chronic stress of managing inadequate resources, the anxiety associated with unpayable energy bills, the shame and social isolation resulting from the inability to maintain comfortable living conditions, and the direct neurophysiological effects of thermal discomfort on mood regulation and cognitive function [6]. The financial strain dimension is particularly significant, as Jacques-Aviñó et al. (2022) documented how anxiety, stress, and distress associated with unpayable energy bills substantially contribute to the mental health burden of energy poverty [7]. This financial stress operates alongside and often compounds with the direct physiological effects of thermal inadequacy, creating cumulative health impacts that exceed the sum of individual stressors.

Whilst much research has focused on cold-related health impacts, the challenge of maintaining adequate cooling during hot weather is increasingly recognised as critical, particularly in Southern European contexts. These findings are particularly relevant for Mediterranean regions where both heating in winter and cooling in summer represent significant challenges, yet research in these contexts remains limited compared to Northern European studies focused primarily on cold-related impacts.

Therefore, there is limited evidence examining how distinct dimensions of energy poverty—thermal inadequacy (i.e., heating and cooling), financial strain related to energy costs, disproportionate energy expenditure and hidden energy poverty—relate to specific physical and mental health conditions within vulnerable populations. This study seeks to address these gaps by presenting a comprehensive analysis of the associations between energy poverty and health outcomes in a Valencia (Spain) sample, specifically examining relationships between multiple energy poverty indicators and a broad range of health outcomes, employing the social determinants of health as a theoretical framework. This approach provides a conceptual foundation, recognising that the conditions in which people are born, grow, live, work and their access to power, money and resources exert powerful influences on health inequities [8]. The theoretical approach of the study is expanded with the integration of Dahlgren and Whitehead’s Social Ecological Model (1991) [9]. This model maps the relationship between individuals, their environment and their health, recognising that people’s well-being is influenced by factors working at multiple levels: constitutional factors, individual lifestyle factors, community influences, living and working conditions, and general socioeconomic, cultural and environmental conditions [9]. This ecological perspective is essential for understanding how energy poverty operates within broader structures of social, economic and political inequality to produce health outcomes.

The research pursues the following specific objectives: O1. to characterise the study sample regarding sociodemographic variables and energy poverty parameters/indicators; O2. to assess self-reported physical and mental dimensions of the sample, prevalence of chronic diseases and use of health services; O3. to analyse existing associations between energy poverty parameters and the evaluated health variables, considering possible relationships with sociodemographic variables.

By responding to the referred objectives, this research provides evidence on how different dimensions of energy poverty affect specific health outcomes among vulnerable populations, informing both theoretical understanding and intervention development to address this critical public health challenge.

## 2. Materials and Methods

### 2.1. Ethical Approval

Ethical procedures were followed before the recruitment and inclusion of participants in the Valencia study. The study protocol is registered as a clinical trial under ISRCTN14905838. The date of registration is 21 February 2022.

### 2.2. Study Design

This study is framed within the WELLBASED project that follows a multisite pretest-posttest control group design to evaluate interventions addressing energy poverty across six European cities.

For the present study, only baseline data from the Valencia pilot site in Spain were analysed. Data collection was conducted between 24 October 2022 and 14 July 2023. This cross-sectional analysis examines the relationship between energy poverty and health outcomes prior to any intervention, providing foundational evidence on energy poverty prevalence and its associations with physical and mental health among Valencia’s vulnerable populations.

#### 2.2.1. Recruitment

A convenience sampling strategy was employed for participant recruitment, leveraging pre-existing relationships between vulnerable households and their key trusted contacts (e.g., social workers, volunteers, NGO staff). These intermediaries were essential for reaching participants, as they could validate the project and support the introduction of the WELLBASED initiative. A referral protocol for potential participants was established between social organisations and WELLBASED staff, ensuring data protection and privacy throughout the process. Face-to-face meetings and direct communication methods were prioritised throughout the recruitment process to build trust-based relationships and overcome digital exclusion and scam concerns among vulnerable households.

#### 2.2.2. Participants

Inclusion criteria:

Adults (>18 y.o.) who are in situation of higher vulnerability, particularly those struggling to pay energy bills or living in poorly insulated homes with inadequate indoor temperatures. This includes, among other individuals who are unemployed, have low income, single parents, parents with dependent children or elderly family members, seniors (over 65) with dependency or health conditions, seniors (over 65) living alone, people with disabilities receiving aids from the municipal social services, young students and members of minority groups (such as Roma community).

Exclusion criteria:

- Individuals who have already been beneficiaries of similar energy poverty interventions;
- Individuals with limitations preventing adequate participation in the WELLBASED intervention (e.g., intellectual disabilities, inability to attend workshops, training or meetings; very poor health conditions, severe language limitations preventing minimal communication, the inability to fully understand the informed consent form required for participation in the project, which outlines their rights and guarantees of anonymization, among other crucial data protection issues);
- Individuals living in households illegally connected to the electricity grid;
- Individuals living in homes requiring large-scale interventions on the structure of the building, or homes requiring major works and/or investment by the family;
- No signed consent form to participate.

#### 2.2.3. Evaluation Measures

Sociodemographic data was collected through an ad hoc questionnaire to provide comprehensive insights into participants’ profiles, whilst additional contextual variables were gathered to capture relevant characteristics directly related to the research objectives.

Health, well-being and quality of life outcomes were collected using validated instruments. Health-related quality of life (HR-QoL) was measured using the EuroQol-5 dimensions (EQ-5D-5L) instrument [10]. This widely used standardised tool assesses HR-QoL across five key dimensions: mobility (ability to walk and move around); self-care (ability to wash and dress oneself); usual activities (ability to perform routine tasks such as housework, family responsibilities, or leisure activities); pain/discomfort (presence and severity of physical discomfort); and anxiety/depression (presence and severity of psychological distress). Complementary health outcomes were measured using two additional validated instruments. The Short Form-12 (SF-12) was employed to assess HR-QoL, specifically examining how chronic conditions impact patients’ perceived quality of life [11]. Mental health outcomes were evaluated using the Depression Anxiety Stress Scale-21 (DASS-21) [12], which measures negative emotional states of depression, anxiety and stress.

Healthcare utilisation patterns were assessed using the modified Self-Management Resource Centre (SMRC) Healthcare Utilisation Questionnaire [13], which captures comprehensive information regarding the frequency and duration of hospital admissions and other healthcare service usage.

Energy poverty status was assessed using the primary indicators developed by the EPAH [14], providing a standardised approach to identifying and characterising energy poverty amongst study participants.

Table A1 presents a comprehensive overview of all outcome measures employed in this study, which were integrated into the WELLBASED self-reported questionnaires. To ensure methodological rigour, validated translations were utilised when available. For instruments lacking validated translations, a systematic back-translation method was employed to ensure cultural adaptation and linguistic accuracy.

#### 2.2.4. Data Analysis

Statistical analyses were carried out using IBM SPSS Statistics (version 30.0). Sociodemographic characteristics and study variables (energy poverty status and health and well-being outcomes) are summarised using descriptive statistics. Additionally, bivariate associations between variables were examined using appropriate statistical tests based on variable types. Pearson correlation coefficients were calculated to assess relationships between continuous sociodemographic variables and continuous health outcomes. One-way analysis of variance (ANOVA) was employed to examine differences in continuous health variables across categorical sociodemographic groups and energy poverty indicators. Chi-square tests of independence were used to evaluate associations between categorical energy poverty indicators and categorical health variables. Statistical significance was set at *p* < 0.05 for all analyses.

## 3. Results

### 3.1. Characterisation of the Sample

#### 3.1.1. Sociodemographic Characteristics

A total of 322 participants (69.6% women) (*M*_age_ = 48, *SD* = 14.145) were included in the study, with 10.9% of participants older than 65 years. Regarding marital status, 52.8% were single, separated, divorced or widowed, whilst 47.2% were married.

The educational level of participants showed a relatively balanced distribution, with 32.0% having completed tertiary education, 35.4% having completed secondary education, 24.8% having completed primary education, and 7.8% having no formal education.

Concerning household income, most participants (46.6%) reported a net monthly household income of up to 750, followed by 21.1% with an income between €751 and €1000.

In terms of employment status, 53.4% of participants had no paid work, whilst 21.4% were employed, 10.2% of respondents and their partners were employed, and 9.3% relied on their partner’s employment. The vast majority (83.8%) lived with someone else, whilst 11.5% lived alone.

Regarding migration background, 63.4% of participants were born in Spain, and 36.6% in another country. Concerning housing, 93.4% resided in apartments, with 51.4% renting at market rate and 19.7% as owners. Table A2 provides an overview of sociodemographic data.

#### 3.1.2. Energy Poverty Status

The energy poverty status of the participants is shown in Table 1.

The findings revealed significant challenges in maintaining adequate home temperatures, with 69.9% of participants reporting an inability to keep their home adequately warm during winter and 72.4% experiencing difficulties maintaining adequate cooling during summer.

Financial difficulties with energy costs were also prevalent, as evidenced by 43.2% of participants declaring arrears on utility bills. Indicators based on energy expenditure (EP4, EP5) showed different patterns: whilst only 8.1% of the population demonstrated low energy expenditure relative to their income (Hidden Energy Poverty or M/2 indicator), a substantial 31.7% showed disproportionate energy expenditure in relation to their income (2M Energy Poverty indicator).

### 3.2. Health, Well-Being and Quality of Life

#### 3.2.1. Health-Related Quality of Life

The assessment of HR-QoL showed differing levels of impairment across the five dimensions (Table 2). These values indicate that pain/discomfort represents the most significant health challenge for participants (*M* = 2.10, *SD* = 1.09), followed by anxiety/depression (*M* = 1.77, *SD* = 1.09), whilst self-care abilities were largely unproblematic (*M* = 1.24, *SD* = 0.60).

#### 3.2.2. Chronicity

The chronic disease burden among participants was considerable, with only 17.1% reporting no chronic conditions, 39.1% having one to two conditions, and 43.8% having three or more conditions (Table 3). This pattern indicates that over four-fifths of participants (82.9%) were managing at least one chronic condition, with pain-related disorders and cardiovascular conditions being the most prevalent (see Table A3 for detailed disease distribution).

#### 3.2.3. Mental Health

The results in Table 4 show that anxiety is the most common mental health condition reported by the participants, with nearly half of the participants (46.3%) exhibiting some level of anxious symptoms. This is followed by stress, with 42.5% of participants showing some level of stress symptoms, and depression, with 36.6% of participants showing some level of depressive symptoms. Notably, severe to extremely severe symptoms were reported by 20.1% for anxiety, 15.2% for stress, and 8.7% for depression.

#### 3.2.4. Use of Health Resources

Healthcare utilisation patterns over the previous 6 months revealed a clear hierarchy in service use. Primary care services were most frequently used, with most participants (81.4%) consulting a physician at least once in the past 6 months, with 42.9% requiring 3 or more visits. Emergency department utilisation was reported by 41.6% of participants, with most frequent users (31.7%) attending accident and emergency (A&E) services 1–2 times. In contrast, hospital admissions were relatively uncommon, affecting only 11.4% of participants, with the majority of these (9.8%) having brief encounters (1–2 admissions) and short stays (87.8% did not spend a night in the hospital, and amongst those hospitalised, most stays were under a week) (see Table A4).

### 3.3. Health and Sociodemographic Characteristics

#### 3.3.1. Relationship Between Health Indicators, Age and Income

The results in Table 5 demonstrate that age presents significant moderate positive correlations with all dimensions of the EQ-5D-5L, except for the anxiety and depression dimension. The strongest correlation is observed with pain and discomfort (*r* = 0.40, *p* < 0.001), followed by mobility problems (*r* = 0.37, *p* < 0.001), difficulties in usual activities (*r* = 0.36, *p* < 0.001), and self-care problems (*r* = 0.35, *p* < 0.001). Whilst there exists a significant but modest correlation between age and anxiety (*r* = 0.14, *p* < 0.05), the correlations between age and depression, stress, and general anxiety/depression did not reach statistical significance (all *p*s > 0.05). Furthermore, age shows a significant but weak correlation with the number of medical visits in the past six months (*r* = 0.24, *p* < 0.001); however, there is no significant correlation with accident and emergency visits, hospitalisations or hospital nights (all *p*s > 0.05), suggesting that the greater use of services is concentrated in primary care and routine medical follow-up rather than severe episodes.

The correlations between health and household income, when significant, are consistently negative: higher household income is only associated with lower levels of anxiety/depression (*r* = −0.32, *p* < 0.001), depression (*r* = −0.23, *p* < 0.05), and anxiety (*r* = −0.24, *p* < 0.01).

#### 3.3.2. Relationship Between Health Indicators and Gender

Regarding mobility, the results in Table 6 indicate that women reported significantly greater difficulties (*M* = 1.67, *SD* = 0.95) compared to men (*M* = 1.44, *SD* = 0.76), *p* = 0.004, η^2^ = 0.04, indicating a small effect size.

For self-care, women also reported slightly more difficulties (*M* = 1.25, *SD* = 0.64) than men (*M* = 1.20, *SD* = 0.49), *p* = 0.010, η^2^ = 0.03, though the effect size was small. This dimension captures challenges with washing and dressing oneself.

Concerning usual activities (e.g., work, study, housework, family or leisure activities), women reported significantly greater impairment (*M* = 1.43, *SD* = 0.84) compared to men (*M* = 1.27, *SD* = 0.59), *p* = 0.027, η^2^ = 0.02, with a small effect size.

Women also experienced significantly more pain and discomfort (*M* = 2.21, *SD* = 1.12) than men (*M* = 1.85, *SD* = 0.94), *p* = 0.005, η^2^ = 0.03, representing a small effect. These findings reflect that women in this sample experienced more limitations in their physical health compared to men.

Mental health measures revealed more pronounced gender differences, with moderate effect sizes. For anxiety/depression (EQ-5D-5L dimension), women reported significantly higher levels (*M* = 1.89, *SD* = 1.13) compared to men (*M* = 1.45, *SD* = 0.85), *p* < 0.001, η^2^ = 0.06, indicating a medium effect size.

The DASS-21 subscales confirmed these patterns with larger effects. Women reported significantly higher depression scores (*M* = 8.98, *SD* = 8.45) than men (*M* = 4.45, *SD* = 5.88), *p* < 0.001, η^2^ = 0.10, representing a moderate effect. Similarly, anxiety scores were substantially higher for women (*M* = 10.30, *SD* = 8.99) compared to men (*M* = 5.55, *SD* = 5.99), *p* < 0.001, η^2^ = 0.09. Stress levels followed the same pattern, with women scoring higher (*M* = 15.74, *SD* = 9.95) than men (*M* = 10.06, *SD* = 7.29), *p* < 0.001, η^2^ = 0.10, also indicating a moderate effect.

Finally, women reported a significantly higher number of chronic illnesses (*M* = 2.82, *SD* = 2.33) compared to men (*M* = 2.36, *SD* = 2.26), *p* = 0.002, η^2^ = 0.03, which reflects that women in this vulnerable sample experienced greater chronic disease burden compared to men.

#### 3.3.3. Relationship Between Health Indicators and Household Composition

The results in Table 7 reveal statistically significant differences between people living alone (*n* = 37) and those living with others (*n* = 270) across multiple health dimensions.

Regarding mobility, participants living alone reported significantly more limitations (*M* = 2.08, *SD* = 1.10) compared to those living with others (*M* = 1.55, *SD* = 0.86), *p* < 0.001, η^2^ = 0.04, representing a small effect size.

For self-care, those living alone also experienced significantly more difficulties (*M* = 1.49, *SD* = 0.77) than those living with others (*M* = 1.21, *SD* = 0.58), *p* = 0.010, η^2^ = 0.02, indicating a small effect.

Usual activities showed the most pronounced difference among physical health dimensions. Participants living alone reported substantially more impairment (*M* = 1.89, *SD* = 0.88) compared to those living with others (*M* = 1.33, *SD* = 0.75), *p* < 0.001, η^2^ = 0.06, representing a small-to-medium effect size. This was the largest effect observed across physical health outcomes, suggesting that difficulties in performing daily activities are particularly marked among people living alone.

Concerning pain and discomfort, participants living alone reported significantly higher levels (*M* = 2.62, *SD* = 1.19) compared to those living with others (*M* = 2.05, *SD* = 1.05), *p* = 0.002, η^2^ = 0.03, indicating a small effect.

The single-item anxiety/depression dimension from the EQ-5D-5L revealed a significant difference, with participants living alone reporting higher levels (*M* = 2.27, *SD* = 1.37) compared to those living with others (*M* = 1.70, *SD* = 1.03), *p* = 0.003, η^2^ = 0.03, representing a small effect.

Interestingly, the more detailed DASS-21 subscales showed different patterns. For depression, although participants living alone showed slightly higher scores (*M* = 8.38, *SD* = 8.05) than those living with others (*M* = 7.51, *SD* = 8.23), the difference was not statistically significant, *p* = 0.547, η^2^ = 0.00. Similarly, for anxiety, those living alone presented higher scores (*M* = 11.51, *SD* = 9.93) than those living with others (*M* = 8.67, *SD* = 8.44), but the difference only approached significance, *p* = 0.061, η^2^ = 0.01, with a small effect size. No statistically significant difference was found for stress (*p* = 0.925, η^2^ = 0.00), with nearly identical scores between groups (living alone: *M* = 14.16, *SD* = 9.42; living with others: *M* = 14.00, *SD* = 9.82).

A statistically significant difference emerged for the number of chronic illnesses, with participants living alone reporting substantially higher chronic disease burden (*M* = 3.78, *SD* = 2.74) compared to those living with others (*M* = 2.59, *SD* = 2.27), *p* = 0.004, η^2^ = 0.03, representing a small effect.

#### 3.3.4. Relationship Between Health Indicators and Bills Support

For receipt of financial support toward energy bills (whether from government or local communities for energy consumption; *n* = 9.9% receiving support, 86.6% not receiving support), no significant associations were found with any of the health variables examined (all *p*-values 0.119–0.940).

### 3.4. Health and Energy Poverty

#### 3.4.1. Relationship Between Continuous Health Indicators and Energy Poverty Indicators

The analysis of continuous health variables in relation to energy poverty indicators presented in Table 8 reveals that:

The EP1 indicator, measuring the ability of participants to keep their homes adequately warm in winter, showed significant associations with several health outcomes. No significant differences were found for mobility, self-care, or usual activities (all *p*s > 0.050). Participants unable to maintain adequate warmth in winter reported significantly higher pain/discomfort (*M* = 2.18, *SD* = 1.09) than those able to maintain adequate warmth (*M* = 1.92, *SD* = 1.06), *p* = 0.044, η^2^ = 0.05. Participants unable to maintain warmth showed significantly elevated anxiety/depression (*M* = 1.87, *SD* = 1.13) compared to those able to do so (*M* = 1.54, *SD* = 0.95), *p* = 0.011, η^2^ = 0.06, indicating a small-to-medium effect.

This pattern was also observed for the three DASS-21 scales. Participants experiencing heating inadequacy in winter reported significantly higher depressive symptoms (*M* = 8.92, *SD* = 8.45), compared to participants able to maintain adequate heat in winter (*M* = 4.84, *SD* = 6.63), *p* < 0.001, η^2^ = 0.11, representing a medium effect. They also experienced significantly more anxiety symptoms (*M* = 10.10, *SD* = 8.93) than those without heating inadequacies (*M* = 6.20, *SD* = 7.04), *p* < 0.001, η^2^ = 0.10, indicating a medium effect. Finally, they reported significantly higher stress symptoms (*M* = 15.45, *SD* = 9.77) than those without heating inadequacies (*M* = 10.90, *SD* = 8.71), *p* < 0.001, η^2^ = 0.10, also representing a medium effect.

The EP2 indicator, measuring the ability to keep homes adequately cool in summer, demonstrated significant associations across multiple health outcomes. Participants unable to maintain adequate cooling during summer presented significantly greater self-care difficulties (*M* = 1.29, *SD* = 0.67) compared to those able to do so (*M* = 1.11, *SD* = 0.35), *p* = 0.019, η^2^ = 0.06, indicating a small-to-medium effect. No other significant differences were found for physical health outcomes (all *p*s > 0.05).

Participants experiencing cooling inadequacy reported significantly higher anxiety/depression scores (*M* = 1.88, *SD* = 1.13) than those not experiencing cooling inadequacies (*M* = 1.49, *SD* = 0.92), *p* = 0.005, η^2^ = 0.07, representing a small-to-medium effect. The pattern for mental health outcomes extended across all three DASS-21 scales with medium effect sizes. Participants unable to maintain adequate cooling reported significantly higher depressive symptoms (*M* = 8.71, *SD* = 8.59) than those able to maintain adequate cooling (*M* = 5.03, *SD* = 6.16), *p* < 0.001, η^2^ = 0.09, indicating a medium effect. They also reported significantly higher anxiety symptoms (*M* = 9.80, *SD* = 8.85) than those not experiencing such inadequacies (*M* = 6.69, *SD* = 7.43), *p* = 0.004, η^2^ = 0.07, representing a small-to-medium effect. Finally, they reported higher stress symptoms (*M* = 14.95, *SD* = 9.64) than those without cooling issues (*M* = 11.88, *SD* = 9.47), *p* = 0.011, η^2^ = 0.06, indicating a small-to-medium effect.

Participants with arrears energy bills (EP3) consistently reported worse outcomes across multiple health outcomes. They showed significantly greater mobility difficulties (*M* = 1.76, *SD* = 0.98) than those without unpaid energy bills (*M* = 1.50, *SD* = 0.83), *p* = 0.011, η^2^ = 0.06, representing a small-to-medium effect. Additionally, they reported more difficulties performing usual activities (*M* = 1.50, *SD* = 0.90) than those without unpaid energy bills (*M* = 1.30, *SD* = 0.67), *p* = 0.025, η^2^ = 0.05, indicating a small effect, and significantly more pain/discomfort (*M* = 2.30, *SD* = 1.10) than those without arrears (*M* = 1.95, *SD* = 1.10), *p* = 0.004, η^2^ = 0.07, representing a small-to-medium effect. There was no significant difference in self-care between groups (*p* > 0.05). In terms of mental health outcomes, there was no significant difference in Hr-QoL anxiety/depression between those experiencing arrears and those who were not (*p* > 0.05). However, participants experiencing arrears reported significantly higher depressive symptoms (*M* = 9.75, *SD* = 8.68) than those not experiencing arrears (*M* = 6.13, *SD* = 7.37), *p* < 0.001, η^2^ = 0.10, indicating a medium effect. They also reported significantly higher anxiety symptoms (*M* = 10.71, *SD* = 8.82) than those without arrears (*M* = 7.59, *SD* = 8.17), *p* = 0.001, η^2^ = 0.08, representing a small-to-medium effect. Interestingly, despite elevated depression and anxiety, participants with arrears did not report significantly higher stress symptoms than those not experiencing arrears (*p* = 0.159)**.**

Participants with arrears also reported a significantly greater burden of chronic diseases (*M* = 3.08, *SD* = 2.39) than those without arrears (*M* = 2.40, *SD* = 2.27), *p* = 0.010, η^2^ = 0.06, indicating a small-to-medium effect.

The EP4 indicator, representing low absolute energy expenditure or “hidden energy poverty,” showed no significant associations with any physical or mental health variables examined (all *p*s > 0.05).

The EP5 indicator, measuring a high share of energy expenditure relative to income, demonstrated selective but meaningful associations with health outcomes. Participants spending disproportionate amounts on energy relative to their income reported significantly more mobility problems (*M* = 1.77, *SD* = 0.99 vs. *M* = 1.55, *SD* = 0.83), *p* = 0.048, η^2^ = 0.05, representing a small effect. They also experienced increased pain/discomfort (*M* = 2.35, *SD* = 1.10 vs. *M* = 2.02, *SD* = 1.10), *p* = 0.013, η^2^ = 0.07, indicating a small-to-medium effect, and higher anxiety/depression scores (*M* = 2.00, *SD* = 1.20 vs. *M* = 1.64, *SD* = 1.10), *p* = 0.008, η^2^ = 0.07, also representing a small-to-medium effect.

For more detail, Table 9 presents the relationship between the individual items of the DASS-21 and the energy poverty indicators.

#### 3.4.2. Relationship Between Categorical Health Variables and Energy Poverty Indicators

The analysis of associations between specific chronic conditions and energy poverty indicators revealed meaningful patterns (See Table 10).

For respiratory conditions, inability to maintain adequate warmth during winter (EP1) demonstrated a marginally significant association (*p* = 0.051, η = 0.109), with 81.6% of participants with lung disease unable to keep their homes warm compared to 18.4% who maintained comfortable temperatures. No other energy poverty indicator was associated with respiratory conditions (all *p*s > 0.05).

Cardiovascular and metabolic conditions showed significant associations with specific energy poverty dimensions. High blood pressure demonstrated a relationship with summer cooling inadequacy (EP2) (*p* = 0.014, η = 0.14), affecting 81.6% of those unable to maintain cool conditions. No other energy poverty indicator was associated with high blood pressure (all *p*s > 0.05).

Musculoskeletal conditions presented significant associations with both thermal and financial dimensions of energy poverty. Osteoarthritis demonstrated a significant association with cooling inadequacy (EP2) (*p* = 0.048, η = 0.11), with 16.7% of participants with osteoarthritis experiencing cooling inadequacy, while the remaining 83.3% did not experience cooling inadequacy. It also demonstrated significant associations with utility bill arrears (EP3) (*p* = 0.044, η = 0.11), with 55.6% of participants with osteoarthritis having utility bill arrears, while the remaining 44.4% did not have utility bill arrears. No other energy poverty indicator was associated with osteoarthritis (all *p*s > 0.05). Back pain showed an association with arrears (EP3) (*p* = 0.008, η = 0.15), affecting 49.2% of those with payment difficulties, while the remaining 50.8% of those with back pain did not have payment difficulties. No other energy poverty indicator was associated with back pain (all *p*s > 0.05). Rheumatoid arthritis demonstrated the strongest association observed with arrears (EP3) (*p* < 0.001, η = 0.21), with 66.7% of individuals with this condition experiencing payment difficulties compared to 33.3% without arrears.

Depression showed significant associations with three primary indicators: winter warmth inadequacy (EP1) (*p* = 0.001, η = 0.18), summer cooling inadequacy (EP2) (*p* = 0.007, η = 0.15), and utility bill arrears (EP3) (*p* = 0.018, η = 0.13). Notably, 83.5% of individuals with depression were unable to maintain adequate thermal comfort in both winter and summer, whilst 54.1% experienced arrears. Other energy poverty indicators were not associated with depression (both *p*s > 0.05).

Blood disease demonstrated a significant association with arrears (EP3) (*p* = 0.034, η = 0.12), affecting 54.3% of those with payment difficulties. Other energy poverty indicators were not associated with blood disease (all *p*s > 0.05).

Interestingly, stomach disease showed a significant association with hidden energy poverty (EP4/M2) (*p* = 0.021, η = 0.14), with 20.7% of those with extremely low energy expenditure reporting this condition compared to 7.8% without hidden energy poverty. Other energy poverty indicators were not associated with stomach disease (all *p*s > 0.05).

#### 3.4.3. Relationship Between Healthcare Utilisation and Energy Poverty Indicators

No significant associations were observed between healthcare utilisation patterns and energy poverty indicators (all *p*-values ranging from 0.060–0.950).

## 4. Discussion

The findings from this study in Valencia provide compelling evidence for the complex relationship between energy poverty and health outcomes, aligning with and extending the existing literature, whilst highlighting associations observed within this particular urban context. These findings can be understood through the framework of Dahlgren and Whitehead’s Social Ecological Model [9], which positions living and working conditions—including housing and energy access—as critical intermediate determinants of health that mediate between structural socioeconomic factors and individual health outcomes.

Our Valencia sample is characterised by 69.9% of participants being unable to maintain adequate winter warmth and 72.4% experiencing summer cooling difficulties. Given that approximately 40 million Europeans (9.3% of the EU population) were unable to keep their homes adequately warm in 2022 [1], and that 33% of the Spanish population experiences energy poverty related to extreme heat [15], it would be reasonable to expect that difficulties in maintaining adequate home temperatures could be extrapolated to these broader populations.

Thus, the study reveals significant challenges in maintaining adequate indoor temperatures year-round, with summer cooling poverty emerging as an especially acute problem in Valencia’s vulnerable populations. This likely reflects both the poor energy efficiency of much of Spain’s building stock [16] and the concentration of low-income households in thermally inadequate housing [17].

Beyond these characterisation patterns, our results demonstrate significant associations between energy poverty indicators and physical health outcomes, particularly with pain/discomfort and mobility. The observed relationship between inadequate winter warmth and increased pain/discomfort is consistent with extensive evidence documenting associations between cold housing and musculoskeletal problems: Liddell and Morris [3] found that inadequate heating was associated with greater pain perception and reduced physical functioning in chronic pain conditions, whilst the Marmot Review Team [4] identified correlations between cold homes and arthritis exacerbation. These associations found in our study indicate that energy poverty co-occurs with diminished physical functioning and reduced daily quality of life in vulnerable populations.

Similarly, our findings reveal a co-occurrence between insufficient summer cooling and cardiovascular conditions, particularly hypertension. This pattern aligns with research documenting relationships between heat exposure and cardiovascular health: Åström et al. [18] found increased cardiovascular mortality during heat exposure among vulnerable populations, while Benmarhnia et al. [19] observed elevated blood pressure during heat waves.

Our analysis also revealed associations between musculoskeletal conditions, specifically, rheumatoid arthritis and energy bill arrears. This association may reflect a complex relationship whereby chronic inflammatory conditions co-occur with both heightened vulnerability to cold-related symptom exacerbation (associated with higher heating needs) and reduced employment capacity, factors that are themselves associated with financial vulnerability [20].

The associations observed with mental health outcomes represent another notable precedent in our findings. Participants unable to maintain adequate winter warmth showed substantially elevated stress responsiveness, including pronounced associations with panic episodes and stress over-reactivity. These patterns align with growing evidence documenting relationships between thermal inadequacy and mental health: Liddell and Guiney [6] identified significant associations between cold housing and various mental health indicators. The magnitude and consistency of mental health associations observed in this study are worth careful interpretation. While energy poverty indicators demonstrated small-to-medium effect sizes that might be characterised as modest in absolute terms, their practical significance should be evaluated considering: the consistency across multiple indicators (winter heating inadequacy, summer cooling inadequacy, and utility arrears all showed similar patterns) and the clinical meaningfulness across mental health domains. For instance, participants experiencing winter heating inadequacy showed *DASS-21 Depression* scores averaging 4.08 points higher than those maintaining adequate warmth, and *DASS-21 Stress scores* averaging 4.55 points higher than those without heating inadequacy. Particularly, changes of 4 points or more in DASS-21 subscales can shift individuals from “normal” to “mild” or “moderate” categories, representing clinically relevant changes associated with increased risk of psychosocial deterioration and reduced quality of life [21,22]. From a public health perspective, even modest individual-level effect sizes can translate into substantial collective burden when affecting large proportions of vulnerable populations, particularly for energy poverty, representing a modifiable risk factor: unlike many mental health determinants, it can be addressed through policy interventions and support programmes, such as those implemented in the WELLBASED project.

Specifically, *DASS-21: Depression* showed consistent associations across three energy poverty indicators: winter warmth inadequacy, summer cooling inadequacy, and utility bill arrears. This consistency across both thermal and financial dimensions is compatible with conceptual models proposing multiple pathways through which energy poverty and mental health may be related, including both physiological responses to thermal discomfort and psychological responses to financial strain [6].

Furthermore, the particularly strong associations between *DASS-21: Anxiety* and energy poverty indicators may reflect the chronic uncertainty and threat associated with energy poverty. Such uncertainty manifests concretely in the “heat or eat” dilemma identified by Day et al. [23], a recurring choice that creates sustained psychological distress.

Beyond specific health dimensions, participants with utility bill arrears reported significantly more chronic conditions overall, suggesting a broader pattern of health-energy poverty co-occurrence. Tod et al. [24] documented how chronic disease is associated with increased energy needs (including heating, powered medical equipment, and greater time spent at home), factors that may increase vulnerability to energy poverty. Research has also documented associations between energy poverty and disease trajectories, with cold stress co-occurring with worsened cardiovascular and respiratory conditions, heat stress associated with metabolic disorder exacerbation, and financial stress related to reduced capacity for disease management [25].

These patterns align with broader frameworks on health inequities. The WHO European Region’s Health Equity Status Report (2019) noted that over 70% of inequities in self-reported health status associated with living conditions co-occurred with housing and energy deprivation [26]. Our findings contribute granular evidence consistent with these population-level patterns, demonstrating how specific energy poverty dimensions are associated with health outcomes and supporting the conceptualization of energy poverty as an important social determinant of health embedded within the broader architecture of health inequities.

Within this broader context, the substantial differences observed between participants living alone and those cohabiting are worthy of attention. Individuals living alone reported markedly higher chronic disease burden, mobility difficulties, and challenges with usual activities. These patterns align with literature identifying solo living as associated with more severe energy poverty outcomes [27]. Solo living co-occurs with multiple vulnerabilities: single-person households face higher per capita energy costs [28], which may be associated with greater vulnerability to energy poverty-related health impacts. From a socio-ecological perspective, household composition represents a critical intermediate layer in understanding how broader structural conditions and individual health outcomes are related, though the mechanisms underlying these associations require further investigation.

Gender differences observed in our sample, with women reporting significantly higher levels across most physical and mental health measures, reflect well-documented patterns in the literature. Bouzarovski and Petrova [29] identified that women experience disproportionate energy poverty impacts due to spending more time at home. These gender disparities illustrate how social and cultural norms—another layer in Dahlgren and Whitehead’s model—interact with material conditions to produce differential health vulnerabilities.

In contrast to these consistent patterns, the absence of significant health associations with extremely low energy expenditure (EP4:M/2 indicator) is noteworthy. This “hidden energy poverty” indicator showed no significant relationships with health outcomes in our analysis. The interpretation of this finding requires caution, given several methodological considerations. The small proportion of participants meeting this criterion in our sample (8.1%) limited statistical power to detect associations. Additionally, as Hills [30] and Boardman [31] have noted in their analyses of energy expenditure data, this indicator may capture heterogeneous household situations that require different interpretations—ranging from efficient energy use to harmful under-consumption. Our cross-sectional design and sample characteristics do not allow us to distinguish between these possibilities. Further research into larger samples and mixed-methods approaches would be needed to clarify the health implications of extremely low energy expenditure in vulnerable populations.

Another notable pattern in our findings concerns healthcare utilisation. Despite substantial health differences associated with energy poverty status, primary care, emergency department, and hospital utilisation showed no significant variations across energy poverty dimensions in our Valencia sample. This likely reflects Spain’s universal healthcare system, which maintains relatively equitable access despite socioeconomic disparities [32].

All these findings have implications for energy poverty policies in Valencia. Our observed associations between energy poverty and health outcomes across physical, mental, and chronic disease domains are consistent with conceptual frameworks positioning energy poverty as a social determinant of health operating at multiple layers of the social ecological model. Addressing these complex, interconnected relationships requires integrated policy responses that recognise both the multidimensional nature of energy poverty and the multiple pathways through which it may be associated with health outcomes. Accordingly, it is recommended to use a Health in All Policies (HiAP) approach when designing public policies to address energy poverty.

Before discussing future research directions, it is important to acknowledge the contextual factors that may affect the transferability of these findings to other settings. The Mediterranean climate is characterised by hot, dry summers with temperatures frequently exceeding 35 °C and mild, relatively short winters [33]. This climatic pattern contrasts sharply with Northern European contexts, where energy poverty research has traditionally focused on winter heating inadequacy due to prolonged cold seasons, creating concentrated winter health risks [5]. In this context, Northern European energy poverty interventions have prioritised winter heating infrastructure improvements such as insulation and heating system upgrades, while summer cooling has historically received minimal attention in both research and policy [6]. In contrast, our findings show a dual burden of heating and cooling inadequacy in Valencia, with both dimensions showing significant health associations. This bidirectional thermal stress creates year-round vulnerability requiring intervention approaches that balance heating and cooling, distinguishing them from Northern European models.

These climatic differences suggest that while the fundamental relationships between energy poverty and health, including thermal stress, financial strain, and social factors, may be relevant across contexts, their relative importance and temporal patterning likely vary considerably across climatic zones. Consequently, our findings may have the greatest direct applicability to other Southern European contexts (southern Spain, southern France, coastal Italy and Greece).

Some limitations warrant acknowledgement. Convenience sampling through social service networks introduces potential selection bias that may limit generalizability. Our sample likely overrepresents individuals already engaged with social services and underrepresents those avoiding formal support systems or lacking connections to intermediary organisations. While we acknowledge this selection bias limits sample representativeness, the substantial variability observed in health outcomes remains informative for examining associations between energy poverty and health outcomes.

Evaluation approaches also merit consideration. All measures relied on self-report, which may introduce recall bias (particularly for healthcare utilisation over the 6-month period) and social desirability bias (especially for sensitive information such as income, energy poverty indicators, and mental health symptoms). To mitigate these limitations, we employed validated instruments with established psychometric properties and used standardised timeframes for recall periods. However, the potential for underreporting of socially stigmatised conditions (e.g., energy poverty, mental health issues) or overreporting of healthcare use should be considered when interpreting findings.

The study design also has some limitations. The cross-sectional design has inherent limitations, preventing causal inference; longitudinal, quasi-experimental or randomised studies are needed to establish temporal relationships between energy poverty and health outcomes.

Regarding our analytical approach, bivariate analyses were deemed appropriate for this pilot study’s exploratory objectives, which aimed to identify initial patterns and associations to inform future hypothesis-driven research. While multivariate analyses adjusting for potential confounders (age, gender, income) would strengthen causal inference, they were not conducted in this baseline analysis due to the primary objective of documenting associations among energy poverty and health outcomes. We acknowledge that this decision increases the risk of Type II error, mitigated by the identification of relevant associations commonly assumed but scarcely evidenced in the literature.

Beyond these limitations, the findings pave the way to further research delving into causality, evaluating whether interventions targeting energy poverty produce health improvements commensurate with observed associations.

## 5. Conclusions

This study in Valencia documents significant associations between energy poverty and health outcomes amongst vulnerable populations.

Sociodemographic patterns revealed important disparities requiring targeted responses. Individuals living alone demonstrated markedly elevated health burdens across multiple domains. Women reported significantly higher levels across most physical and mental health measures, reflecting their disproportionate exposure to domestic thermal conditions. Notably, healthcare utilisation showed no significant variations by energy poverty status despite substantial health differences, likely reflecting Spain’s universal healthcare system maintaining equitable access. However, this service equalisation should not obscure the persistent health burdens created by energy poverty.

The findings demonstrate that energy poverty is associated with multiple dimensions of both physical and mental health. Mental health associations were particularly pronounced, with participants unable to maintain thermal comfort exhibiting substantially elevated depression, anxiety, and stress across validated measures. Physical health associations revealed condition-specific patterns: respiratory conditions were associated with heating inadequacy, cardiovascular conditions (particularly hypertension) co-occurred with cooling inadequacy, and musculoskeletal conditions showed strong associations with utility bill arrears.

From a public health perspective, these findings underscore that addressing energy poverty may represent an essential component of health equity strategies. Urban contexts with Mediterranean climate patterns present unique challenges requiring year-round interventions encompassing both winter heating and summer cooling, moving beyond traditional cold-weather focus.

## Figures and Tables

**Table 1:** Characterisation of participants’ energy poverty status based on EPAH primary indicators (*n* = 322).

Energy Poverty Indicators	*n* (%)
(EP1)—Inability to keep home adequately warm in winter	225 (69.9%)
(EP2)—Inability to keep home adequately cold summer	233 (72.4%)
(EP3)—Arrears on utility bills	139 (43.2%)
(EP4)—Low absolute energy expenditure (M/2)	26 (8.1%)
(EP5)—High share of energy expenditure in income (2M)	225 (69.9%)

**Table 2:** Health-related quality of life dimensions (*n* = 322).

EQ-5D-5L	Level 1–No Problem	Level 2–Slight Problems	Level 3–Moderate Problems	Level 4–Severe Problems	Level 5–Unable to/Extreme Problems	*M* (*SD*)
Mobility	200 (62.1%)	68 (21.1%)	34 (10.6%)	20 (6.2%)	0 (0%)	1.61 (0.91)
Self-care	268 (83.2%)	36 (11.2%)	14 (4.3%)	3 (0.9%)	1 (0.3%)	1.24 (0.60)
Usual activities	244 (75.8%)	43 (13.4%)	26 (8.1%)	7 (2.2%)	2 (0.6%)	1.39 (0.78)
Pain/Discomfort	116 (36.0%)	109 (33.9%)	51 (15.8%)	40 (12.4%)	6 (1.9%)	2.10 (1.09)
Anxiety/Depression	182 (56.5%)	74 (23.0%)	35 (10.9%)	20 (6.2%)	11 (3.4%)	1.77 (1.09)

**Table 3:** Chronic disease (*n* = 322).

Number of Chronic Conditions	*n* (%)
0	55 (17.1%)
1–2	126 (39.1%)
3 or more	140 (43.8%)

**Table 4:** Mental health and well-being (*n* = 322).

DASS-21	*n* (%)
Depression	
Normal	204 (63.4%)
Mild	48 (14.9%)
Moderate	42 (13.0%)
Severe	15 (4.7%)
Extremely severe	13 (4.0%)
Anxiety	
Normal	173 (53.7%)
Mild	27 (8.4%)
Moderate	51 (15.8%)
Severe	28 (8.7%)
Extremely severe	43 (13.4%)
Stress	
Normal	185 (57.5%)
Mild	43 (13.4%)
Moderate	45 (14.0%)
Severe	37 (11.5%)
Extremely severe	12 (3.7%)

**Table 5:** Correlations between health indicators and age and household income (*n* = 322) (Pearson’s correlations rounded to two decimal places).

Health		Age	Net Monthly Household Income
Mobility	Pearson’s correlation (*r*)	0.37	−0.07
	Sig.	<0.001	0.425
	*N*	322	121
Self-care	Pearson’s correlation (*r*)	0.35	−0.02
	Sig	<0.001	0.850
	*N*	322	121
Usual activities	Pearson’s correlation (*r*)	0.36	−0.04
	Sig.	<0.001	0.671
	*N*	322	121
Pain/discomfort	Pearson’s correlation (*r*)	0.40	−0.11
	Sig.	<0.001	0.217
	*N*	322	121
Anxiety/depression	Pearson’s correlation (*r*)	0.10	−0.32
	Sig.	0.076	<0.001
	*N*	322	121
DASS-21: Depression	Pearson’s correlation (*r*)	0.10	−0.23
	Sig.	0.087	0.011
	*N*	322	121
DASS-21: Anxiety	Pearson’s correlation (*r*)	0.14	−0.24
	Sig.	0.014	0.008
	*N*	322	121
DASS-21: Stress	Pearson’s correlation (*r*)	0.003	−0.13
	Sig.	0.592	0.158
	*N*	322	121
No. of physician visits in past 6 months	Pearson’s correlation (*r*)	0.24	−08
	Sig.	<0.001	0.385
	*N*	322	12
No. of A&E visits in past 6 months	Pearson’s correlation (*r*)	−0.00	−0.17
	Sig.	0.971	0.064
	*N*	322	121
No. of different hospital stays overnight or longer in the past 6 month	Pearson’s correlation (*r*)	0.09	−0.11
	Sig.	0.096	0.238
	*N*	317	117
No. of total hospital nights in the past 6 month	Pearson’s correlation (*r*)	0.06	−0.17
	Sig.	0.305	0.061
	*N*	320	119

**Table 6:** Differences between men and women on health dimensions (*n* = 321) (There was a single person who preferred not to specify their gender, “I prefer not to say”) (Mean, F, SD and effect size rounded to two decimal places).

Health	Gender	*N*	Mean (*SD*)	F (1319)	Sig.	η^2^
Mobility	Male	97	1.44 (0.76)	9.20	0.004	0.04
	Female	224	1.67 (0.95)			
	Total	321	1.61 (0.91)			
Self-care	Male	97	1.20 (0.49)	3.31	0.010	0.03
	Female	224	1.25 (0.64)			
	Total	321	1.24 (0.60)			
Usual activities	Male	97	1.27 (0.59)	4.36	0.027	0.02
	Female	224	1.43 (0.84)			
	Total	321	1.39 (0.78)			
Pain/discomfort	Male	97	1.85 (0.94)	12.38	0.005	0.03
	Female	224	2.21 (1.12)			
	Total	321	2.10 (1.09)			
Anxiety/depression	Male	97	1.45 (0.85)	23.52	<0.001	0.06
	Female	224	1.89 (1.13)			
	Total	321	1.77 (1.09)			
DASS-21: Depression	Male	97	4.45 (5.88)	2082.20	<0.001	0.10
	Female	224	8.98 (8.45)			
	Total	321	7.70 (8.15)			
DASS-21: Anxiety	Male	97	5.55 (5.99)	2161.60	<0.001	0.09
	Female	224	10.30 (8.99)			
	Total	321	8.94 (8.58)			
DASS-21: Stress	Male	97	10.06 (7.29)	2855.80	<0.001	0.10
	Female	224	15.74 (9.95)			
	Total	321	14.11 (9.68)			
Nº chronic diseases	Male	97	2.36 (2.26)	67.51	0.002	0.04
	Female	224	2.82 (2.33)			
	Total	321	2.70 (2.35)			

**Table 7:** Differences between participants living alone and those living together with at least one person on health dimensions (*n* = 322) (Mean, F, SD and effect size rounded to two decimal places).

Health	Household Composition	*N*	Mean (*SD*)	F (1320)	Sig.	η^2^
Mobility	Living alone	37	2.08 (1.10)	9.11	<0.001	0.04
	Living with family, partner, or caregiver	270	1.55 (0.86)			
	Total	307	1.62 (0.91)			
Self-care	Living alone	37	1.49 (0.77)	2.47	0.010	0.02
	Living with family, partner, or caregiver	270	1.21 (0.58)			
	Total	307	1.24 (0.61)			
Usual activities	Living alone	37	1.89 (0.88)	10.42	<0.001	0.06
	Living with family, partner, or caregiver	270	1.33 (0.75)			
	Total	307	1.39 (0.79)			
Pain/discomfort	Living alone	37	2.62 (1.19)	10.70	0.002	0.03
	Living with family, partner, or caregiver	270	2.05 (1.05)			
	Total	307	2.12 (1.08)			
Anxiety/depression	Living alone	37	2.27 (1.37)	10.58	0.003	0.03
	Living with family, partner, or caregiver	270	1.70 (1.03)			
	Total	307	1.77 (1.09)			
DASS-21: Depression	Living alone	37	8.38 (8.05)	24.48	0.547	0.00
	Living with family, partner, or caregiver	270	7.51 (8.23)			
	Total	307	7.62 (8.20)			
DASS-21: Anxiety	Living alone	37	11.51 (9.93)	262.36	0.061	0.01
	Living with family, partner, or caregiver	270	8.67 (8.44)			
	Total	307	9.02 (8.66)			
DASS-21: Stress	Living alone	37	14.16 (9.42)	0.86	0.925	0.00
	Living with family, partner, or caregiver	270	14 (9.82)			
	Total	307	14.02 (9.75)			
Nº chronic diseases	Living alone	37	3.78 (2.74)	46.17	0.004	0.03
	Living with family, partner, or caregiver	270	2.59 (2.27)			
	Total	307	2.74 (2.36)			

**Table 8:** Relation between EQ-5D-5L, n° chronic diseases and DASS-21 and EP indicators (*n* = 322) (Mean, SD and effect size rounded to two decimal places) (EP4 has been excluded from table because none of the health variables showed significant associations).

Energy Poverty		Mobility	Self-Care	Usual Activities	Pain Discomfort	Anxiety Depression	No. Chronic Diseases	T0_Depress_Dass	T0_Anxiety_Dass	T0_Stress_Dass
EP1: Inability to keep home adequately warm in winter	Yes Mean (SD)	ns	ns	ns	1.92 (1.06)	1.54 (0.95)	2.24 (1.97)	4.84 (6.63)	6.20 (7.04)	10.90 (8.71)
	No Mean (SD)				2.18 (1.09)	1.87 (1.13)	2.89 (2.47)	8.92 (8.45)	10.10 (8.93)	15.45 (9.77)
	F (1, 320)				4.75	7.61	28.67	1127.78	1040.60	1365.65
	Sig.				0.044	0.011	0.022	<0.001	<0.001	<0.001
	η^2^				0.05	0.06	0.05	0.11	0.10	0.10
EP2: Inability to keep home adequately cold in summer	Yes Mean (SD)	ns	1.11 (0.35)	ns	ns	1.49 (0.92)	2.29 (2.11)	5.03 (6.16)	6.69 (7.43)	11.88 (9.47)
	No Mean (SD)		1.29 (0.67)			1.88 (1.13)	2.85 (2.42)	8.71 (8.59)	9.80 (8.85)	14.95 (9.64)
	F (1, 320)		1.98			9.36	20.65	871.54	621,27	605.05
	Sig.		0.019			0.005	0.053	<0.001	0.004	0.011
	η^2^		0.06			0.07	0.05	0.09	0.07	0.06
EP3: Arrears on utility bills	Yes Mean (SD)	1.76 (0.98)	ns	1.50 (0.90)	2.30 (1.10)	ns	3.08 (2.39)	9.75 (8.68)	10.71 (8.82)	ns
	No Mean (SD)	1.50 (0.83)		1.30 (0.67)	1.95 (1.10)		2.40 (2.27)	6.13 (7.37)	7.59 (8.17)	
	F (1, 320)	5.26		3.03	9.75		36.15	1037.64	770.86	
	Sig.	0.011		0.025	0.004		0.010	<0.001	0.001	
	η^2^	0.06		0.05	0.07		0.060	0.101	0.079	
EP5: High share of energy expenditure in income (2M)	Yes Mean (SD)	1.77 (0.99)	ns	ns	2.35 (1.10)	2.00 (1.20)	ns	ns	ns	ns
	No Mean (SD)	1.55 (0.83)			2.02 (1.10)	1.64 (1.10)				
	F (1, 320)	3.27			7.22	8.62				
	Sig.	0.048			0.013	0.008				
	η^2^	0.05			0.07	0.07				

**Table 9:** Relation between individual items of DASS-21 and EP indicators (*n* = 322) (Mean, SD and effect size rounded to two decimal places) (EP4 and some of the DASS-21 items have been excluded from table because no significant associations were found).

Energy Poverty		Difficulty Winding Down	Aware of Mouth Dryness	Cannot Seem to Experience Any Positive Feeling	Difficulty Breathing Without Physical Activity	Tend to Over-React	Experience Trembling	Feeling Worried to Panic or Embarrass Self	Feeling Nothing to Look Forward to	Difficulty Relaxing
EP1: Inability to keep home adequately warm in winter	Yes Mean (SD)	0.94 (1.02)	ns	0.31 (0.57)	0.54 (0.82)	0.53 (0.75)	0.44 (0.74)	0.39 (0.62)	0.36 (0.0.63)	1.02 (1.08)
	No Mean (SD)	1.38 (1.09)		0.62 (0.82)	0.83 (0.94)	1.05 (0.95)	0.68 (0.89)	0.79 (0.83)	0.61 (0.75)	1.31 (1.02)
	F (1, 320)	13.10		6.64	14.39	18.55	3.66	10.57	4.17	5.55
	Sig.	<0.001		<0.001	<0.001	<0.001	0.023	<0.001	0.005	0.024
	η^2^	0.08		0.08	0.06	0.13	0.05	1.06	0.06	0.05
EP2: Inability to keep home adequately cold in summer	Yes Mean (SD)	ns	ns	0.37 (0.63)	0.51 (0.76)	0.65 (0.83)	ns	0.42 (0.65)	0.33 (0.62)	ns
	No Mean (SD)			0.59 (0.80)	0.83 (0.95)	0.98 (0.94)		0.76 (0.83)	0.61 (0.75)	
	F (1, 320)			3.04	6.71	7.06		7.81	5.34	
	Sig.			0.022	0.004	0.004		<0.001	0.001	
	η ^2^			0.05	0.07	0.07		0.09	0.08	
EP3: Arrears on utility bills	Yes Mean (SD)	ns	ns	0.71 (0.84)	0.87 (0.95)	1.01 (0.97)	0.71 (0.89)	0.89 (0.86)	0.68 (0.77)	ns
	No Mean (SD)			0.39 (0.67)	0.64 (0.88)	0.80 (0.88)	0.52 (0.80)	0.50 (0.70)	0.42 (0.67)	
	F (1, 320)			8.31	4.22	3.71	2.78	12.31	5.45	
	Sig.			<0.001	0.024	0.037	0.048	<0.001	0.010	
	η ^2^			0.10	0.05	0.05	0.046	0.117	0.079	
EP5: High share of energy expenditure in income (2M)	Yes Mean (SD)	ns	ns	0.67 (0.80)	ns	1.04 (1.00)	ns	ns	0.63 (0.80)	ns
	No Mean (SD)			0.44 (0.75)		0.81 (0.90)			0.45 (0.67)	
	F (1, 320)			3.28		3.43			2.102	
	Sig.			0.018		0.046			0.043	
	η ^2^			0.06		0.05			0.053	
**Energy Poverty**		**Felt Downhearted/Blue**	**Intolerant of Obstacles**	**Felt Close to Panic**	**Not Enthusiastic About Anything**	**Felt Low** **Self-Worth**	**Felt Rather Touchy**	**Felt Scared Without Reason**	**Felt Life Is Meaningless**	
EP1: Inability to keep home adequately warm in winter	Yes Mean (SD)	0.49 (0.74)	0.42 (0.72)	0.27 (0.56)	0.33 (0.63)	0.25 (0.58)	0.35 (0.78)	0.29 (0.66)	ns	
	No Mean (SD)	0.92 (0.92)	0.70 (0.88)	0.59 (0.76)	0.63 (0.78)	0.44 (0.72)	0.69 (0.98)	0.44 (0.72)		
	F (1, 320)	12.25	5.30	6.88	5.97	2.63	7.76	1.55		
	Sig.	<0.001	0.006	<0.001	0.001	0.018	0.002	0.077		
	η ^2^	0.10	0.07	0.09	0.08	0.06	0.08	0.04		
EP2: Inability to keep home adequately cold in summer	Yes Mean (SD)	0.51 (0.73)	0.38 (0.73)	0.29 (0.51)	0.33 (0.58)	0.19 (0.40)	0.38 (0.73)	0.27 (0.60)	0.10 (0.34)	
	No Mean (SD)	0.90 (0.92)	0.71 (0.87)	0.57 (0.78)	0.62 (0.79)	0.46 (0.75)	0.67 (0.92)	0.44 (0.74)	0.31 (0.63)	
	F (1, 320)	10.08	6.85	4.85	5.50	4.63	7.59	1.91	2.90	
	Sig.	<0.001	0.002	0.002	0.002	0.002	0.003	0.049	0.003	
	η ^2^	0.09	0.08	0.07	0.08	0.08	0.07	0.05	0.07	
EP3: Arrears on utility bills	Yes Mean (SD)	0.99 (0.95)	0.79 (0.94)	0.65 (0.76)	0.71 (0.83)	0.52 (0.61)	1.00 (1.00)	ns	ns	
	No Mean (SD)	0.64 (0.81)	0.49 (0.74)	0.37 (0.67)	0.40 (0.66)	0.28 (0.69)	0.77 (0.87)			
	F (1, 320)	9.18	7.35	6.58	7.49	4.32	4.36			
	Sig.	<0.001	0.010	<0.001	<0.001	0.020	0.025			
	η ^2^	0.084	0.079	0.089	0.092	0.073	0.046			
EP5: High share of energy expenditure in income (2M)	Yes Mean (SD)	ns	ns	ns	ns	ns	ns	ns	ns	
	No Mean (SD)									
	F (1, 320)									
	Sig.									
	η ^2^									

**Table 10:** Relation between individual items of chronicity and EP indicators (Only those relationships between health and EP that show statistically significant differences are presented) (Effect size rounded to two decimal places).

EP Indicator		Disease No		Disease Yes		Total (*N*)	χ^2^	Sig.	η
		*N*	%	*N*	%				
Lung disease									
EP1: Inability to keep home adequately warm in winter	Yes	88	32.2	9	18.4	97	3.80	0.051	0.11
	No	185	67.8	40	81.6	225			
High blood pressure									
EP2: Inability to keep home adequately cold in summer	Yes	71	31.7	18	18.4	89	6.07	0.014	0.14
	No	153	68.3	80	81.6	233			
Osteoarthritis									
EP2: Inability to keep home adequately cold in summer	Yes	80	29.9	9	16.7	89	3.91	0.048	0.11
	No	188	70.1	45	83.3	233			
EP3: Arrears on utility bills	Yes	109	40.7	30	55.6	139	4.06	0.044	0.11
	No	159	59.3	24	44.4	183			
Backpain									
EP3: Arrears on utility bills	Yes	45	34.4	94	49.2	139	7.00	0.008	0.15
	No	86	65.6	97	50.8	183			
Rheumatoid arthritis									
EP3: Arrears on utility bills	Yes	103	38.4	36	66.7	139	14.60	<0.001	0.21
	No	165	61.6	18	33.3	183			
Depression									
EP1: Inability to keep home adequately warm in winter	Yes	83	35.0	14	16.5	97	10.23	0.001	0.18
	No	154	65.0	71	83.5	225			
EP2: Inability to keep home adequately cold in summer	Yes	75	31.6	14	16.5	89	7.20	0.007	0.15
	No	162	68.4	71	83.5	233			
EP3: Arrears on utility bills	Yes	93	39.2	46	54.1	139	5.64	0.018	0.13
	No	144	60.8	39	45.9	183			
Blood disease									
EP3: Arrears on utility bills	Yes	101	40.1	38	54.3	139	4.51	0.034	0.12
	No	151	59.9	32	45.7	183			
Stomach disease									
EP4: Low absolute energy expenditure (M/2)	Yes	20	7.8	6	20.7	26	5.30	0.021	0.14
	No	238	92.2	23	79.3	261			

## Data Availability

The data are not publicly available due to privacy or ethical restrictions.

## Abbreviations

The following abbreviations are used in this manuscript:

EPAH	Energy Poverty Advisory Hub
EP	Energy Poverty
QoL	Quality of Life
HR-QoL	Health-related quality of life
SF-12	Short Form-12
DASS-21	Depression Anxiety Stress Scales-21
SMRC	Self-Management Resource Centre
ANOVA	One-way analysis of variance
Accident and Emergencies	A&E
Health in All Policies	HiAP

## Appendix A

### Outcome Measures Used in the Comprehensive Evaluation of Health and Energy Poverty

**Table A1:** Outcome measures used in the comprehensive evaluation of health and energy poverty.

Outcome	Instruments
Sociodemographic details: age, sex, gender, occupation, etc.	Ad hoc questionnaire
Health and well-being measures	
Qualtiy of Life HRQoL	EQ-5D-5L
Self-perceived health (chronicity)	SF-12 Health Survey (SF12)
Mental health (Depression, anxiety, stress)	DASS-21
Healthcare utilisation	
Care utilisation	SMRC Health Care utilisation questionnaire
Energy poverty status	
Energy poverty indicators	EP1: Inability to keep home adequately warm in winter, EP2: inability to keep home adequately cold in summer, EP3: arrears on utility bills, EP4: low absolute energy expenditure (M/2), EP5: high share of energy expenditure in income (2M)

## Appendix B

### Descriptive Results

**Table A2:** Socio-demographic characteristics (*n* = 322).

Socio-Demographic	
Gender, *n* (%)	
Female	224 (69.6%)
Male	97 (30.1%)
Prefer not to say	1 (0.3%)
Age (years)	
Mean (SD)	48.8 (14.1)
Older people (>65 years)	35 (10.9%)
Marital status, *n* (%)	
Married	152 (47.2%)
Single, separated, divorced or widowed	170 (52.8%)
Educational level, *n* (%)	
No education	25 (7.8%)
Primary education	80 (24.8%)
Secondary education	114 (35.4%)
Tertiary education	103 (32.0%)
Household income category, *n* (%)	
1 (Less or 750 €)	150 (46.6%)
2 (751 € to 1000 €)	68 (21.1%)
3 (1001 € to 1300 €)	51 (15.8%)
4 (1301 € to 1650 €)	25 (7.8%)
5 (1651 € to 2000 €)	9 (2.8%)
6 (2001 € to 2350 €)	6 (1.9%)
7 (2351 € to 2800 €)	2 (0.6%)
8 (2801 € to 3500 €)	4 (1.2%)
9 (3501 € or more)	7 (2.2%)
Paid work, *n* (%)	
Yes, by respondent	69 (21.4%)
Yes, by respondent’s partner	30 (9.3%)
Yes, by respondent and their partner	33 (10.2%)
No	172 (53.4%)
Household composition *	
Living with family, partner, or caregiver	270 (83.8%)
Live alone	37 (11.5%)
Migration background	
Yes	204 (63.4%)
No	118 (36.6%)
Belonging to an ethnic minority	
Yes	10 (3.1%)
No	230 (71.4%)
Prefer not to say/don’t know	82 (25.5%)
Dwelling type *	
Detached/Semi-detached/terraced	4 (1.2%)
Apartment or flat	298 (93.4%)
Other	3 (0.9%)
Tenure status *	
Outright owner/owner paying mortgage	63 (19.7%)
Rent at market rate	164 (51.4%)
Reduced/free rent	55 (17.2%)
Other	23 (7.2%)
Receiving financial support towards energy bills (any source) *	
Yes	32 (9.9%)
No	275 (85.4%)

* 15 missing cases.

**Table A3:** Chronic disease (*n* = 322).

Chronic Conditions	*n* (%)
No. of chronic conditions	
0	55 (17.1%)
1–2	126 (39.1%)
3 or more	140 (43.8%)
Heart Disease	
YES	32 (9.9%)
High blood pressure	
YES	98 (30.4%)
Lung disease	
YES	49 (15.2%)
Diabetes Type I or II	
YES	29 (9.0%)
Ulcer or stomach disease	
YES	29 (9.0%)
Kidney disease	
YES	19 (5.9%)
Liver disease	
YES	12 (3.7%)
Blood disease	
YES	70 (21.7%)
Cancer within last 5 years	
YES	8 (2.5%)
Depression	
YES	85 (26.4%)
Osteoarthritis/generative arthritis	
YES	54 (16.8%)
Back pain	
YES	191 (59.3%)
Rheumatoid arthritis	
YES	54 (16.8%)
Other medical problems	
YES	140 (43.5%)

**Table A4:** Participants’ health care use (*n* = 322).

SMRC Health Care Utilisation	*n* (%)
No. of physician visits in the past 6 months	
0	60 (18.6%)
1–2	124 (38.5%)
3 or more	138 (42.9%)
No. of hospital accident & emergency visits in the past 6 months	
0	188 (58.4%)
1–2	102 (31.7%)
3 or more	32 (9.9%)
No. of different times staying in the hospital overnight or longer in the past 6 months (*n* = 317) *	
0	281 (88.6%)
1–2	31 (9.8%)
3 or more	5(1.6%)
No. of total nights spent in hospital in the past 6 months (*n* = 320) **	
0	281(87.8%)
1–2	22 (6.9%)
3–7	14 (4.5%)
8 or more	3 (0.9%)

* 5 missing cases. ** 2 missing cases.
